# Enhancing public entertainment with touch: Possibilities and pitfalls

**DOI:** 10.1177/20416695241280715

**Published:** 2024-10-18

**Authors:** Charles Spence, Yang Gao

**Affiliations:** Crossmodal Research Laboratory, 150583 Department of Experimental Psychology, University of Oxford, Oxford, United Kingdom

**Keywords:** entertainment, cinema, touch, vestibular, haptics, multisensory

## Abstract

There has long been interest in augmenting cinematic and other forms of public entertainment through tactile and/or bodily (i.e., vestibular) stimulation. In this narrative historical review, the early history of touch (or haptics, as it is sometimes called) and other forms of bodily stimulation (e.g., motion platforms) in the context of entertainment is critically reviewed, with a focus on early cinema as well as other early examples of immersive virtual reality travel experiences. Critically, various challenges have limited the introduction of such additional channels of sensory stimulation. These include technological, financial, cognitive, creative, ethical/artistic, and also legal considerations, given the many patents that currently exist covering commercial digital tactile stimulation (e.g., in the gaming context). Taken together, these challenges help to explain why it is that despite the early interest in “the feelies” (e.g., an envisioning of film that includes tactile sensations by Aldous Huxley, in his novel Brave New World), touch-enhanced cinema and storytelling have never really caught on in the mainstream in the way that, say, the talkies so obviously did following the introduction of sound into cinema in the early decades of the 20th century. Nevertheless, identifying the potential successful use cases that have emerged from previous attempts to augment public entertainments with tactile/bodily stimulation will likely provide useful guidelines for the future tactile augmentation of home entertainment.

“Going to the Feelies this evening, Henry?” enquired the Assistant Predestinator. “I hear the new one at the Alhambra is first-rate. There's a love scene on a bearskin rug; they say it's marvellous. Every hair of the bear reproduced. The most amazing tactual effects.” (Huxley, 1932)^1^

## Introduction

Likely inspired by the Tactilismo movement promoted by the Italian Futurists (e.g., see Marinetti, 1921), Aldous Huxley, in his *Brave New World* (1932), provided one view of the future of cinema (see the above quote; and see Richards, 2010, for the cultural context of 1930s cinema in England). As Grossi (2023, p. 89) notes: “the fictional feely Three Weeks in a Helicopter appears as a parodic pastiche which bears the marks of various cinematic paradigms of the time: especially early ‘cinema of attractions.’” According to Laura Frost: “Huxley's feelies reach backward to cinema's music hall origins and forward to the imagination of technologies such as virtual reality” (Frost, 2006, p. 450).^2^ Their aim is not so much to tell a story, but rather to enchant and attract the spectator through the deployment of the medium's spectacular power. It is interesting to consider whether Huxley's poor eyesight may have influenced his interest in “the feelies” (see Frost, 2006). As Morton Heilig wrote in an article about the future of cinema that was first published in 1955: “Cinerama, Colorama, Panoramic Screen, Cinemascope, Three-D, and Stereophonic Sound. A dozen marquees in Time Square are luring customers into the realm of a ‘sensational new experience’” (Heilig, 1992, p. 279).

There has long been a desire to augment film (and other forms of multisensory entertainment—defined as “audience-centred commercial culture”; McKee et al., 2014) through additional sensory inputs (e.g., Barjavel, 1944; Heilig, 1992; Joe & Gilman, 2010; Pursey & Lomas, 2018). First came sound and the talkies (between 1926 and 1930; Kehr, 2010; though see also Huxley, 1929). Rapidly thereafter, people's interest turned to the possibilities associated with the introduction of scent (Heilig, 1992; Spence, 2021a), touch (Heilig, 1992), and, more recently, taste/flavour (i.e., matching food and drink to cinema experiences; e.g., Jamieson, 2012; Velasco et al., 2018).

Although it has been trialled on multiple occasions, scent-enhanced cinema never caught on in the way that the talkies so obviously did (see Spence, 2020, for a review). As Heilig (1992, p. 282) put it: “It is the addition of sound that represents the really great ‘revolution’ in the history of cinema.” Though even the introduction of sound was not without its concerns. According to Field and Smith (1933, p. 214): “Experiments have shown that the majority of cinema-goers cannot both look and listen. When they go to the pictures, they have the tendency to look, for had they wished to listen, they would have stayed at home and turned on the wireless.” That said, contemporary experimental psychology research clearly demonstrates that, contrary to Field and Smith's early suggestion, people are able to attend to auditory and visual content simultaneously (Spence & Soto-Faraco, 2020; though see Spence & Read, 2003).

Food and drink have recently been integrated into the edible cinema concept (Dean, 2016; Jamieson, 2012; Spence, 2017), yet this has not become widespread. However, the skin, despite being the body's largest sense organ, accounting for something like 18% to 21% of body mass (Montagu, 1971), has never really been targeted effectively in the context of the cinema. As such, Heilig's (1992, p. 284) vision of the cinema of the future where: “The air will be filled with odours and up to the point of discretion or aesthetic function we will feel changes of temperature and the texture of things. We will feel physically and mentally transported into a new world” has never really come to pass. But why not? One of the challenges with augmenting the number of channels of communication (in any situation) is the limitations of tactile information processing, and the attendant dangers of sensory overload (see Gallace & Spence, 2014; Malhotra, 1984; Spence, 2014). In fact, a closer look at the hopes and realities associated with touch-enabled cinema over the last century or so reveals a number of challenges, including those that are technological, financial, cognitive, creative, ethical/artistic, and also, in some cases, legal (given the many patents that have been filed/granted in touch-enabled technology, especially in the context of gaming) in nature.

In this narrative historical review (see Ferrari, 2015; Furley & Goldschmied, 2021, on the relative merits of this style of review), we take a closer look at the role of technologically delivered touch in cinema and broader entertainment experiences contexts. To some commentators (e.g., Faust & Yoo, 2006), it clearly seems surprising that haptics (which has been defined as “engaging the user via the sense of touch”; Murdey, 2006, p. 2; note here that the term is used by psychophysicists to refer specifically to active touch, whereas human–computer interaction researchers typically use the term as a synonym for touch, no matter whether it is active or passive) are not more present in entertainment given that, as Margaret Minsky (2000) wrote at the turn of the century: “Haptics and entertainment are a natural combination because many popular recreations involve the body and all its senses together. Examples of recreation and entertainment that are inherently haptic are travel, sports, amusement parks, dance, crafts, and making music.”

During the middle decades of the 20th century, there was widespread enthusiasm (and also seemingly optimism) concerning the possible use of the skin as an additional channel of communication (e.g., Collins, 1970; Geldard, 1957). For instance, Frank A. Geldard (1957, p. 115), one of the most prominent researchers in the field, wrote that: “Indeed, our eyes and our ears are assaulted so continuously, such frequent and insistent demands are placed on them, that the visual and auditory channels are seriously overburdened at times. Such oversaturation leads quite naturally to the question of whether it is only vision and hearing that can serve in communication.” It is noticeable how those promoting such tactile/haptic technologies in the context of the cinema always seem to be convinced that there is a market for the new technology. Just take the following quote regarding the introduction to touch-activated seating in cinemas: “Matt Eyre, Cineworld's vice president of operations, says 3D is here to stay and claims 4D seats are not a gimmick but a logical progression. ‘This is the next step. I think filmgoers are always looking for that little bit extra’” (Scott, 2013).^3^

As Marianna Obrist, one of the researchers currently working in this area, put it a few years ago: “Sound and vision aren’t always enough. Being able to smell the odours that a character on screen would smell, or feel the objects or atmosphere they would feel can create and anticipation and build suspense in the same way as sound currently does” (as quoted in Dean, 2016). However, from a different point of view, there is far less reason to be optimistic about touch's potential role in cinema. As Heilig (1992, p. 247) noted (in an article that was first published in 1955): “Each basic sense will dominate the scene in roughly the same proportion we found them to have in man. That is, sight, 70%; hearing, 20%; smell, 5%; touch, 4%; and taste, 1%.” Even if one chooses not to take Heilig's suggestions at face value (and, in fact, there is no reason that we should, given that it is unclear on what evidential basis Heilig came up with the percentages he reports), there is plenty of other evidence supporting a similar hierarchy of the senses, at least in terms of their ability to process information, and/or access cortical resources (see Gallace et al., 2012; see Nørretranders, 1991, for an alternative estimate of the bit rate of the senses; see Table 1). That said, just because the skin (or the sense of touch) may lose out to the ears and eyes in terms of attentional capture, or maximum bit rate in terms of information transfer, there is a separate question concerning the affective importance of each of the senses. It has sometimes been suggested that the sense of smell may dominate, and the sense of touch too can carry a hugely important affective value (see Spence, 2022c).

**Table 1.** Table summarising the number of sensors, number of afferents, information transmission rates/channel capacity (from Zimmerman, 1989), % of attentional capture (from Heilig, 1992) and % of neocortex (Felleman & Van Essen, 1991) relative to each sensory modality.

Sensory sytem	No. of sensors	No. of afferents	Channel capacity (bits/s)	Psychophysical channel capacity (bits/s)	% Attentional capture (Heilig, 1992)	% Neocortex
Vision	2 × 10^8^	2 × 10^6^	10^7^	40	70	55
Audtion	3 × 10^4^	2 × 10^4^	10^5^	30	20	3.4
Touch	10^7^	10^6^	10^6^	5	4	11.5
Taste	3 × 10^7^	10^3^	10^3^	1(?)	1	0.5
Smell	7 × 10^7^	10^5^	10^5^	1(?)	5	(?)

Nevertheless, at this point, one has to ask whether people would really be willing to pay a premium for a touch-enabled cinematic experience, given that it contributes so little (at least in terms of peer-reviewed scientific research demonstrating the benefits; see Danieau et al., 2014) to the overall multisensory experience. According to Scott (2013), writing a little over a decade ago: “The seats will add £5.50 to the £8.90 cost of the average 3D ticket. They will form about 10% of the seating in theatres selected for 4D.” A few years later, Jackson (2015) suggested an additional cost of £2 per ticket in Milton Keynes, perhaps hinting at a decline in the premium charged for haptic augmentation. Nevertheless, it must remain an empirical question as to whether the general public deem it worth paying for the augmented experience. Techniques such as Discrete Choice Modelling could perhaps be used to assess how much consumers in different markets would be willing to pay (cf. Califano & Spence, 2024).

Other commentators remain sceptical of the extent to which virtual touch could ever simulate real touch (Dennett, 1991). As Michael Abrash, chief scientist, at Oculus VR put it: “As important as haptics potentially is for VR, it's embryonic right now. There's simply no existing technology or research that has the potential to produce haptic experiences on a par with the real world. So any solution will have to come from breakthrough research.” (quoted in Parisi, 2018, p. 323). There have, though, been a few examples where a tactile element has been successfully introduced into a cinematic (or entertainment) context. Those examples that can perhaps be considered as successful primarily tend to be in those contexts in which the storyline doesn’t change much (e.g., as in theme park rides). But how exactly did the early pioneers of multisensory entertainment experiences engage with their audience's bodies, and stimulate their skin? In the following section, we first take a closer look at how cinema was augmented with touch in the decades following Huxley's prophetic pastiche appearing in his *Brave New World*. Thereafter, we take a closer look at those forms of entertainment that have successfully stimulated the audience's skin/body (see Table 2). This review will not, though, touch (if you’ll excuse the pun) on the way in which film may promote bodily consciousness (or embodiment) in the viewer (Antunes, 2012; Barker, 2009; Marks, 2000, 2002; Ransom, 2021; Sobchack, 2004), nor will it address the links between cinema and tactile aesthetics (Iosifyan et al., 2017), nor the potential use of the autonomous sensory meridian response to elicit bodily sensations via audiovisual media (e.g., see Barratt et al., 2017). Nor, finally, does this review attempt to provide a state-of-the-art as far as currently available tactile stimulation technologies are concerned (as that would likely constitute an entire paper in its own right). Rather, the focus is squarely on evaluating those technology-mediated tactile stimulation solutions that have been trialled in the marketplace over the years.

**Table 2.** A brief history of tactile/bodily stimulation in the context of shared (or public) visual/audiovisual entertainment.

Date	Name of experience	Description
c. 1895	The “Haunted Swing”	Visual-vestibular conflict introduced in the context of fairground entertainment (Wood, 1895)
1900	Maréorama at the 1900 Universal Exhibition in Paris	Massive pistons introduce sway to hugely popular albeit one-off multsensory experience (Hernández Barbosa, 2023)
c. 1900	Hale's Tours	Tilting train carriage and wind effects stimulate the audience's bodily and tactile senses (Fielding, 1970)
1932	Brave New World (Aldous Huxley)	Tacile cinema imagined in a popular novel
1935	The Ghost Train	Fairground ride in which unexpected tactile stimulation (‘danglers') surprises the punters (Spence, 2024)
1959	The Tingler	First actual use of tactile (vibration) in a subset of seats for dramatic effect, and to break the “fourth wall”
1974	Earthquake	Five movies were released that use Sensurround sound to stimulate the audience's bodies using ultralow frequency sounds
1976	Midway	
1977	Rollercoaster	
1978	Battlestar Galactica/Saga of a Star World	
1979	Mission Galactica: The Cylon Attack	
1975	The Rocky Horror Picture Show	Audiences spontaneously engage in multisensory interactive stimulation with water effects, etc. (Austin, 1981)
1977	Kentucky Fried Movie	“Feel-a-Round” experience imagined (see Pozo, 2016)
20th c.	Star Wars, etc.	Expansion of simulation rides in theme parks (Rabinowitz, 2006)
2012	John Carter	Canadian company (D-Box) introduces a number of shaking seats in cinemas in North America and United Kingdom (Pozo, 2016; Vaughn, 2014)
2014	Kingsman: The Secret Service	
2019	Avengers: Endgame	

*Note.* See main text for the limitations affecting the interpretations of these data.

## A Very Short History of Tactile Cinema

Leeder (2011, p. 773) describes how: “A horror film called *The Tingler* came marked with a new technological innovation called ‘Percepto’ that forced audiences to ‘scream for [their] lives’. At key moments of the film (which was released on July 29th, 1959), certain audience members would, however, get jolted by vibrating motors hidden in their seats.” Leeder (2011, p. 791) goes on to highlight that: “Not all moviegoers were able to experience the gimmick. The cost belonged to the theatre, which was free to do it as well or as poorly as budget permitted, with some ignoring the gimmick altogether.” The technology needed to stimulate a few lucky members of the audience was apparently built from vibrators that had been salvaged from World War II aircraft and were activated at random by the projectionist in certain scenes (Browne & Browne, 2001). The exhibitor's manual for the movie called for the projectionist to “‘give two pushes in rapid sequence’ to the vibrating motors under the seats” (Heffernan, 2004, p. 103) at the appropriate point in the movie.

According to Leeder (2011), Castle makes the following speech at the opening of *The Tingler*: “I’m William Castle, the director of the motion picture you’re about to see. I feel obligated to warn you that some of the sensations, some of the physical reactions, which the actors on the screen feel, will also be experienced, for the first time in motion picture history, by some members of this audience. I say certain members because some people are more sensitive to these mysterious electronic impulses than others. These unfortunate sensitive people will at times feel a strange tingling sensation. Others will feel it less strongly. But don’t be alarmed—you could protect yourself. If at any times you are conscious of a tingling sensation, you can obtain immediate relief by screaming. Don’t be embarrassed about opening your mouth and letting rip with all you got. Because the person sitting in the seat right next to you will probably be screaming too. Remember this: a scream at the right time may save your life.”

According to Leeder (2011, p. 774): “Castle's gimmicks attempt to reach out to the audience and incorporate them directly into the cinematic experience, to restore the real or imagined experience of the early cinema spectator.” Note, though, that according to Leeder's (2011) description of events, the tactile stimulation appears to have been introduced at only one point during the film. Specifically, this was at the moment when the audience was pitched into total darkness (as the projector was temporarily stopped). This both helps to address any concerns about visual dominance (Hutmacher, 2019) as well as helping to dissolve the separation between the on-screen action and the space of the auditorium (Leeder, 2011; cf. Fisher, 2007). Note also that touch is typically advantaged under conditions where vision is impaired [e.g., due to blurring (Heller, 1983) or darkness; Facchini & Aglioti (2003); Marx et al., 2003]. Touch has also sometimes been reported to dominate over vision in the perception of fine surface textures (Guest & Spence, 2003; Lederman et al., 1986).

**Figure 1.**
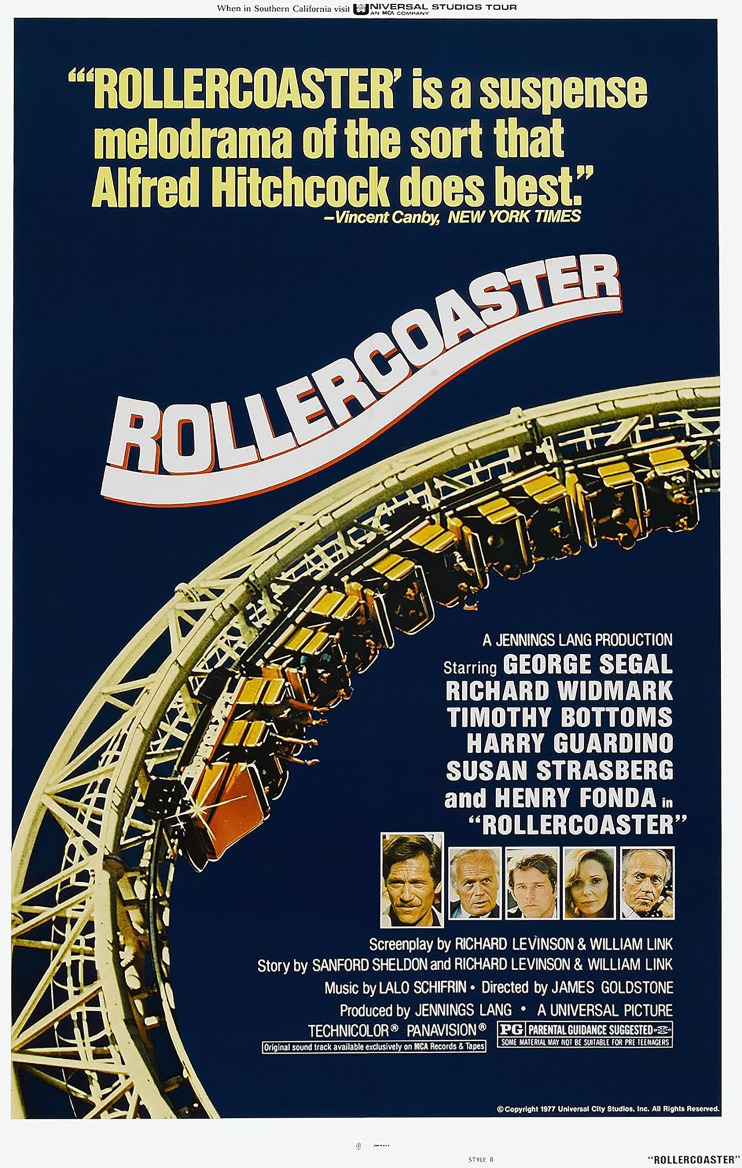
Poster for Rollercoaster movie in Sensurround.

In 1974, a few filmmakers started to experiment with technologies like Sensurround sound, which used ultra-low-frequency rumbles to evoke physical sensations and enhance immersion (Perno, 2015).^4^ Action scenes were accompanied by waves of high decibel sound “[giving] audiences the feeling of being part of the film” (Castle, 1976, p. 247). The same technology was also used in the film *Earthquake* (released November 15th, 1974; see Robson, 1974)^5^ and *Rollercoaster* (June 17th, 1977). In the latter disaster movie, a terrorist went around blowing up amusement park rides. Special subwoofers in select theatres would vibrate the movie seats during select sequences of the film to simulate riding on a rollercoaster (see Figure 1). However, according to Cabanillas (2020), Sensurround was only ever used in a total of five films, and even then presumably only in a small subset of theatres that had been equipped with the relevant speaker technology. Indeed, reviewing the original movie using the technology, *The New York Times* could not imagine it would have any use except in earthquake movies—or, as it termed them, “splat” movies (see Anon, 1974). According to the review: “Sensurround seems to be the last word in audience, participation gimmicks, it beats 3-D, CinemaScope and the smellies by being a kind of elaboration of stereophonic sound to produce what Aldous Huxley, predicted would be the feelies” (Anon, 1974).

According to Anon (2019), the Sensurround system incorporated as many as 20 folded horn-loaded loudspeakers that went as low as 16 Hz, producing volumes up to 120 dB. The practical recorded low-end reached as low as 10 Hz following further refinements (cf. Rottigni & Spence, 2024). These frequencies cannot be heard, but they can be felt. “Theatres had to rent the special equipment designed and built by Cerwin-Vega and BGW Amplifiers for $2000. The first two rows of seats, as well as seats in each of the rear corners needed to be removed making the format an expensive proposition for theatre owners. Despite the obstacles, when the movie *Midway* was released in 1976 in a slightly less aggressive version of the format, there were around 800 Sensurround theatres in the US.”

In the black comedy film *Kentucky Fried Movie* (August 10th, 1977), director John Landis envisioned a “Feel-a-Round” experience for cinema with tactile engagement. Instead of relying on new technologies, it was imagined that moviegoers would be massaged by ushers sitting behind them, and providing them with a hands-on experience: for example, massaging the audience during romantic scenes, synchronising with the action seen on the screen (Pozo, 2016). Ironically, it is precisely this sort of warm interpersonal touch (and stroking) that is now known to deliver the emotional benefits associated with the stroking of the C-Tactile afferent system (see Spence, 2022c). It was, however, never realised, perhaps unsurprisingly given growing public concern about unwanted interpersonal touch over the last half century or so (see Field, 2001). That said, the incorporation of physical touch in a mixed reality (MR) setting has been trialled in both artistic and technical settings (e.g., see Desnoyers-Stewart et al., 2024; Fisher, 2007; Pells, 2015).

Canadian company D-Box released an advertisement for their “motion seats” in 1992 and added a “motion code” track for hundreds of traditional films.^6^ Seats could vibrate, tilt, pitch roll, and heave according to the timing of the scenes (Pozo, 2016). This new device was promoted as a technological utopia: “feel it all” and “Moving the World with Haptic Technology” (D-Box, 1998–2024). The moving seats provided a subset of the audience with new sensations on their skin and body, potentially adding extra meaning to the film and creating a more memorable multisensory experience. However, if the goal of multisensory films is to immerse audiences more deeply and for them to empathise with the protagonists as the director intends, then the design may need to be more carefully thought out. For instance, the D-Box version of *Kingsman: The Secret Service* (Vaughn, 2014) used vibrations to depict violent onscreen action, causing the audience to identify with the characters whose heads had just exploded. This resulted in a less humorous interpretation of the film and apparently left some viewers feeling unsettled (Pozo, 2016). This example hints at the ethical/artistic challenge concerning whether or not it is appropriate to modify the filmmaker's intentions, by choosing to add tactile stimulation post-production. Note that the question of whether it is appropriate to modify the experience as intended by the creator has also been noted in the context of enhanced art experiences (cf. Pursey & Lomas, 2018; Vi et al., 2017).

Although it is often claimed that augmenting cinematic experience with more senses will enhance a viewer's immersion in film (e.g., Danieau et al., 2014), the opposite has also sometimes been reported. For instance, Colin Ransom (2021, p. 44) writes of how: “When I attended a screening of Anthony and Joe Russo's *Avengers: Endgame* (Russo & Russo, 2019), due to high demand, the only available seats were in 4DX. These seats were a new experience for me. They incorporated many ‘effects,’ such as chair motions and vibrations, sprays of mist, fragrant scents, and even the ever-perplexing back and leg tickler. While the goal of 4DX is to provide a more immersive moviegoing experience, I noticed myself less immersed in the film and more attentive and anticipative to the various stimuli around me.”

In Glasgow, the first 4D cinema experience involved a screening of the Disney action film *John Carter* (Bradshaw, 2012; Scott, 2013). According to the press, most of the 3,000 existing D-Box seats are to be found in North America, with others in Japan, Germany, Australia, and New Zealand. The individual controls for each seat can be turned up, down, or off entirely—though it is hard to imagine anyone who has paid a premium for such an experience doing the latter (except perhaps while trying to drink or eat their popcorn). According to one press report, there were plans to introduce “4D” vibrating chairs in thirty cinemas across the United Kingdom (Scott, 2013). Meanwhile, writing in *The New York Times*, Barnes (2014) suggested that the technology may help to lure the young back to the cinema.^7^ However, Philip Drake, of Stirling University's school of film and media studies, noted that while 4D seats were an interesting idea, they were unlikely to become widespread and replace traditional seating given the not-inconsiderable costs associated with retrofitting cinemas (as discussed in Scott, 2013; though see also Thornhill, 2019). In other words, while the filmmakers’ pursuit of tactile sensation in film continues, it is neither widely used nor seemingly especially popular in cinema currently (see Table 3). In conclusion, except in the context of the VR-simulators that one often finds in amusement parks (about which, more below), there has seemingly been little appetite to enable tactile stimulation in the context of commercial cinema. At the same time, it should be noted that no practical system has been introduced for filmmakers to augment the films they make with a tactile track, in the same way as was done, albeit briefly, in the context of scent-enabled cinema (see Spence, 2020).

**Table 3.** Considerations/challenges associated with the augmentation of public audiovisual entertainment/storytelling involving the stimulation of the skin/bodily senses.

Challenge	Description
Technological	What kinds of tactile stimulation is it technically possible to deliver, and to which skin sites?
Financial	Are customers willing to pay for the costs associated with purchase, installation, and maintenance of dedicated tactile stimulation technology?
Cognitive	Assuming that the technical and financial issues are resolved, do the audience possess sufficient cognitive bandwidth, such that the addition of an extra channel of tactile/haptic stimulation delivers sufficient benefit to the overall multisensory experience (see Gallace et al., 2012)?
Creative	Are there industry-standard protocols that allow for the control/delivery of digital tactile stimulation? How to ensure that tactile augmentation goes beyond the merely pleonastic (cf. Banes, 2001).
Ethical/artistic	Is it acceptable to modify the director's/creative's intentions by adding tactile stimulation postproduction that change a work's meaning?

## On the Use of Bodily Stimulation in the Context of Public Entertainment

Around 1900, a range of entertainments emerged briefly that engaged the sense of touch and/or the body (i.e., proprioception/vestibular). These included everything from the craze for electrotactile machines, where people would pay to receive an electric shock (see Parisi, 2013, 2018, 2019),^8^ to the *Maréorama* multisensory virtual travel experience in Paris in 1900, and Hale's Tours in the years around 1900 in London and North America. The first report of the “Haunted Swing” illusion also appeared in print in 1895 at the San Francisco State Fair (Wood, 1895; as well as appearing in Hopkins, 2020, book of magic first printed in 1898). The latter visual-vestibular illusion (Spence, 2022a; Wade & Hughes, 2002) created a range of unusual bodily sensations by rocking the swinging platform on which the public were stationed, while independently rotating the room itself.

### The Maréorama in the 1900 Universal Exhibition

**Figure 2.**
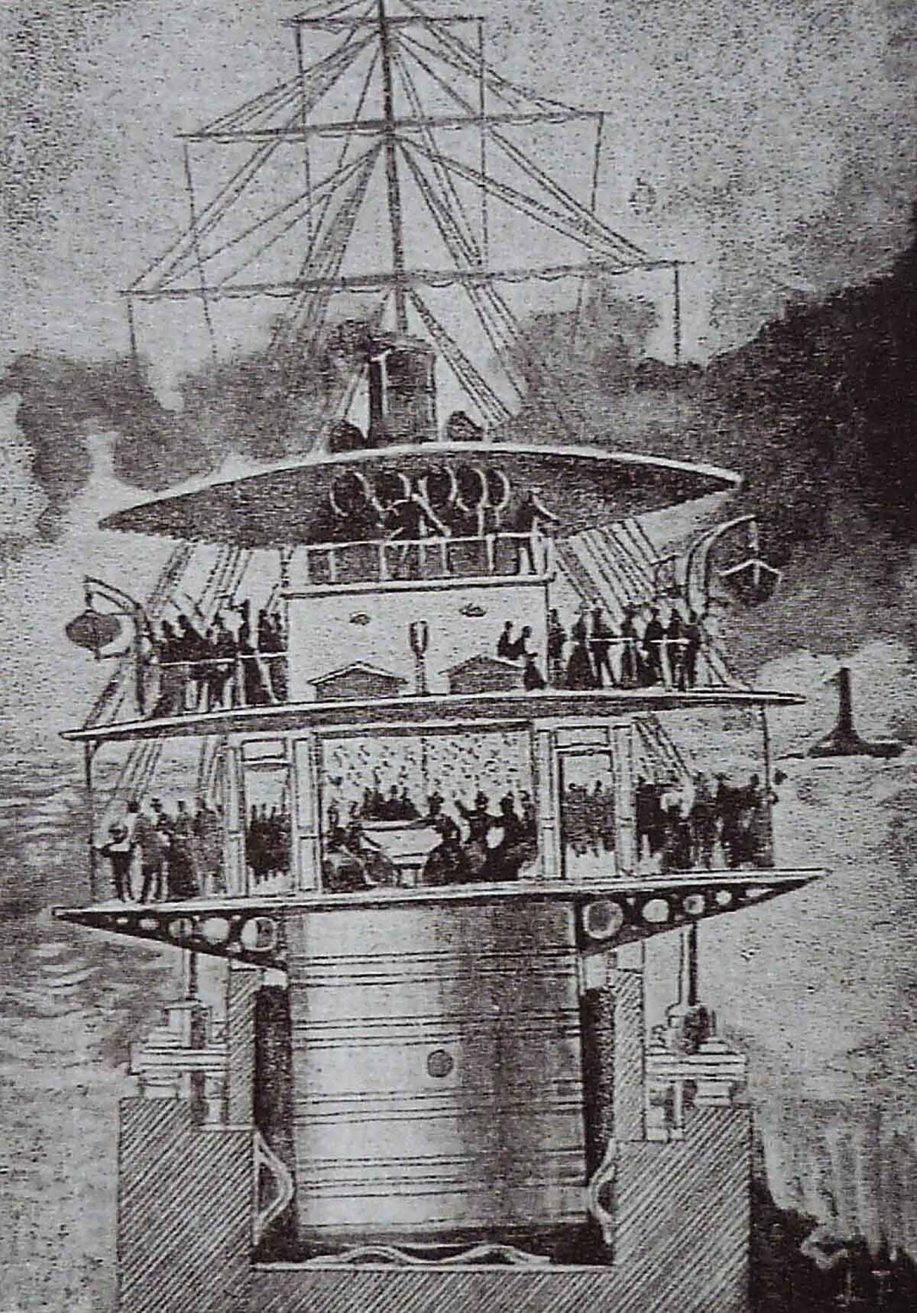
Design of the *Maréorama* showing the mobile feature of the platform. Archive historique de la ville de Paris. Documents éphémères. Exposition universelle de 1900.

*Source:* Reprinted from Hernández Barbosa (2023).

In Paris, the multisensory panorama known as the *Maréorama* offered audiences the experience of a half-hour boat trip across the Mediterranean as part of the hugely successful 1900 Universal Exhibition in Paris (Hernández Barbosa, 2015, 2023). The device itself, a ship-shaped mobile panorama situated by the Seine, offered the public a form of technological entertainment (Sastre-Juan & Valentines-Álvarez, 2016). Huge canvases (c. 1000 × 13 m) painted by the engineer and artist Hugo d’Alési, depicting various Mediterranean scenes, were slowly moved past either side of the ship-shaped platform (33 × 7 m) on which the audience was standing (which itself also moved thus affecting the passengers’ sense of balance). According to Hernández Barbosa (2023, p. 352): “The platform was installed on top of four hydraulic pistons and a Cardan suspension, which was based on a series of concentric rings with orthogonal axes” (see Figure 2). As many as 1,500 “passengers” could be accommodated on this platform in separated first- and second-class seating compartments.

In fact, the physical shocks generated by the motion of the supporting boat-shaped platform apparently led some audience members to feel seasick (Quantin, 1900, p. 352). It is reported that the crew also operated a number of devices generating different multisensory effects, including lights, wind, and the release of sea smells. According to Hernández Barbosa (2023, p. 352): “the innovative nature of the Maréorama was rooted in the explicit and simultaneous appeal to different senses: images, sounds, feelings (which affect motion and balance) and smells.” A simulated storm with lightning and thunder was also included. Meanwhile, an invisible orchestra played live music that was in some sense congruent with the country where the cruise was supposed to be passing by (Anon, 1900, p. 122). Hernández Barbosa (2023, p. 352) writes of how: “the *Maréorama* created a sensation of motion and space perceived directly through the body. The appeal to these physical bodily effects, both in terms of vestibular sense—which places the body in space—and kinaesthetic sense, insofar as the device imitated the body's motion through space (Gubern, 1999, p. 158), presented the audience with experiences that replicated those undergone during a real sea voyage.”

However, albeit being very popular with the general public (indeed it is estimated that the Paris exhibition received over 50 million visitors; Ory, 1982, p. 153), this would appear to have been a one-off experience,^9^ and was soon replaced by Hale's Tours and its imitators (see below). Much like a number of the other successful entertainments that have targeted the body, the spectators on the *Maréorama* were, in some sense, turned into actors who participate actively in the action. As Hernández Barbosa (2023, p. 361) notes: “In this regard, the aim was in no case to encourage the audience ‘to sit in rows in relative silence and to regulate their responses and interactions’ (Nead, 2007, p. 25) as the experience of movie-going is understood today, but for the spectators to engage with the show in a freer and more unpredictable way.” Such a description would seem equally apposite in the case of *The Rocky Horror Picture Show* or other immersive theatre productions such as those popularised in recent years by the likes of the Punchdrunk immersive theatre company (Rose, 2012).

### Hale's Tours

Hale's Tours (sometimes referred to as Hale's Tours and Scenes of the World, Hale's Tours Cars of the World, etc.; Fielding, 1970; Huhtamo, 2012, pp. 318–319) offered a virtual travel experience in which the passengers would enter a phantom train carriage (Hayes, 2009; Rabinovitz, 1998b, 2004, 2006). Robert Paul, the early film pioneer, imagined something of the sort inspired by H. G. Wells’ *The Time Machine* in an 1896 interview published in *The Era* (Anon., 1896): “In a room capable of accommodating some hundred people, he would arrange seats to which a slight motion could be given. He would plunge the apartment into Cimmerian darkness, and introduce a wailing wind. Although the audience actually moved but a few inches, the sensation would be of travelling through space. From time to time the journey would be combined with panoramic effects. Fantastic scenes of future ages would be shown first. Then the audience would set forth upon its homeward journey. The conductor would regretfully intimate that he had over-shot the mark, and travelled into the past—cue for another series of pictures.”

Hale's Tours was introduced commercially at the 1904 St. Louis Exposition. In a train carriage, the projection recording from the front of the train was shown. On occasion, an artificially produced rush of air was presented, and the whole car might pivot on its longitudinal axis so that the operator could, by throwing a lever, sway the car from side to side during the show (hence presumably engaging both the sense of touch and the bodily senses). Later, however, with the increasing sophistication of the design, many of these manual operations were accomplished electrically (see Keefe, 1904, for the original patent application). As well as the sight of the train racing down the track, the tilting of the carriage when the train was seen going round a bend, and the wind blown on the audience's face when the train picked up speed, there was an auditory accompaniment.

**Figure 3.**
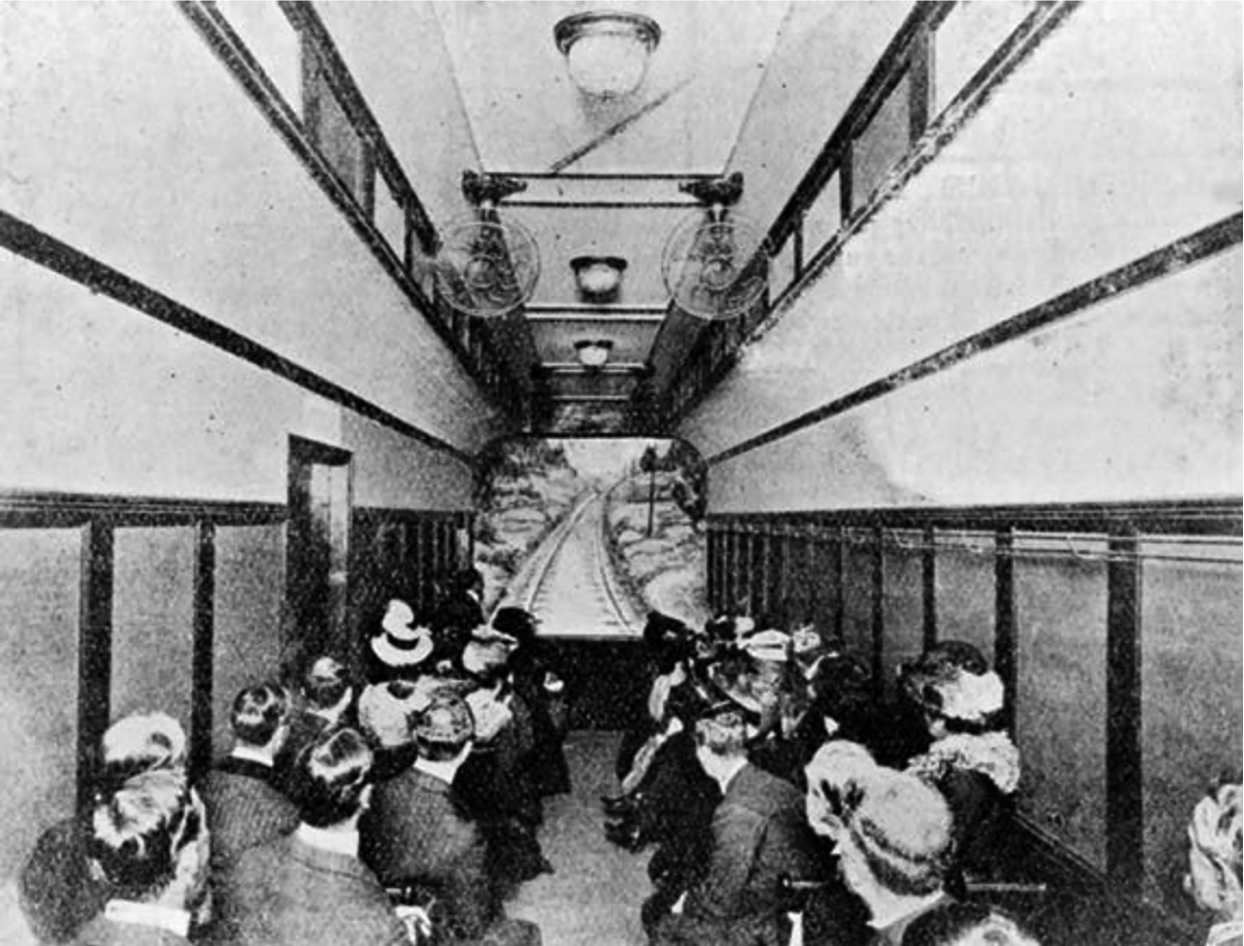
Interior of one of Hale’s Tours.

*Source:* Figure appears in Curtis and Voss (2008).

Rabinovitz (1998a, pp. 146–147) writes of how: “In Hale's Tours installations, the railway car theatre was rocked from side to side, steam whistles tooted, and the sound of wheels clattering was made in order to simulate railroad travel and to foreground the body itself as a site for sensory experience” (see Figure 3). At the time, one finds press reports suggesting that: “The rocking has caused the women to remain away after the first visit (Variety, September 22nd, 1906, p. 11)” (as quoted in Fielding, 1970, p. 46). Nevertheless, as Rabinovitz (2006, p. 60) notes, as early as its initial 1906 season, *Hale's Tours* and its competitors became top-grossing, popular concessions across the United States (Anon, 1906, p. 28). As Rabinovitz (2006, p. 59) notes: “*Hale's Tours* had many competitors of either rail or auto travel: *Palace Touring Cars*, *Hurst's Touring New York*, *Cessna's Sightseeing Auto Tours* … *Hruby and Plummer's Tours and Scenes of the World* made these concepts more generic for travelling carnivals so that they could set up a train, boat, or automobile.” By the end of the 1906 summer season, there were more than 500 installations at amusement parks and storefront theatres in all major U.S. and Canadian cities as well as in capitals of Europe (The Billboard, 1906). However, the lack of new material soon led to the disappearance of this early form of virtual reality (VR; i.e., within 20 years of its introduction).

### Participatory Cinematograph: The Cinematic Shooting Gallery

**Figure 4.**
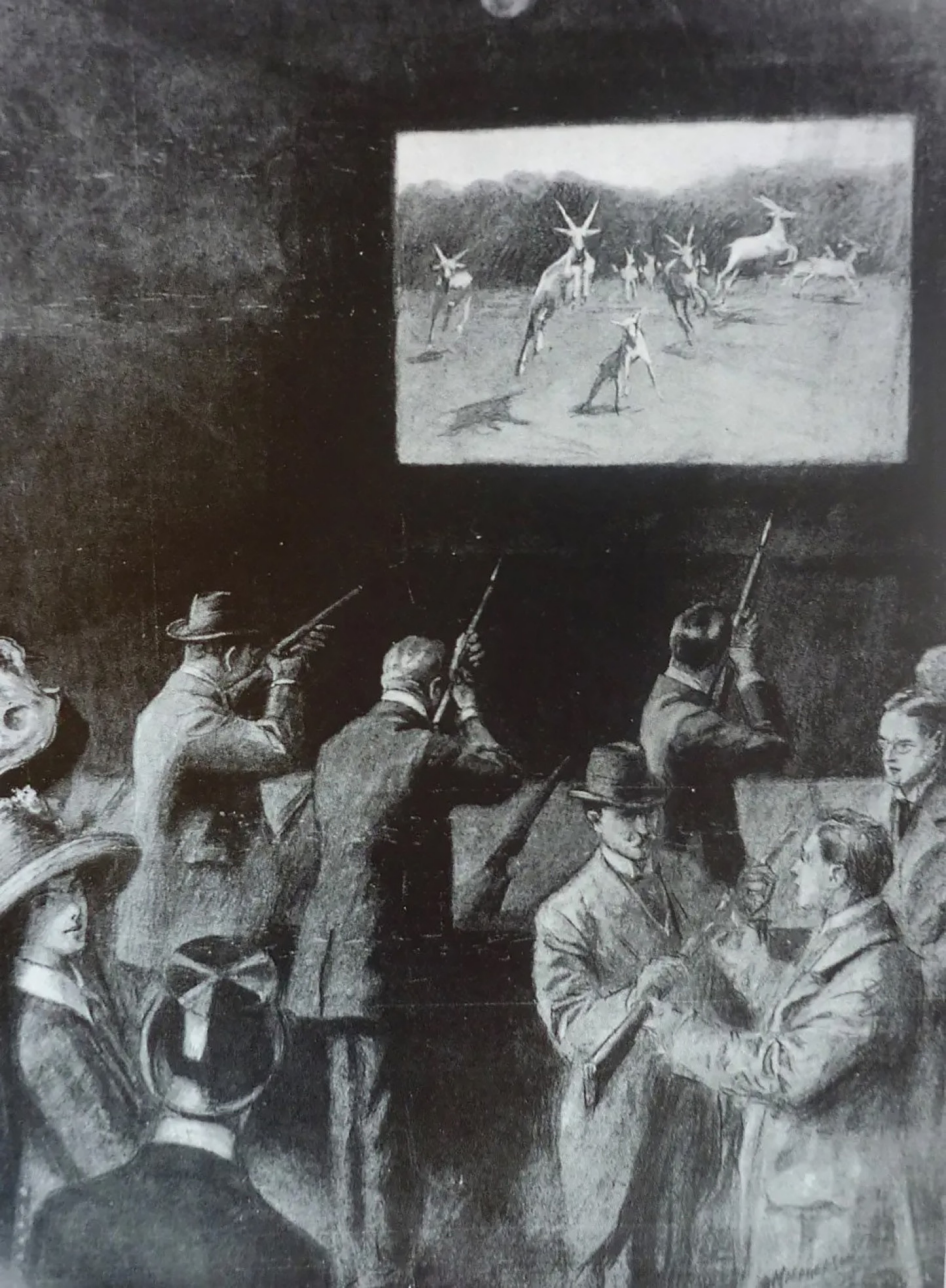
“Sport on the Cinematograph. A new use for moving pictures.”

*Source: The Graphic*, July 26, 1913.

*“*Big Game Hunting by Cinematograph: How to bag rhinoceros in the parlour. BIG-GAME HUNTERS will be glad to know that they can have all the sport of the chase in their own homes.” So read an article that appeared in *The World's Fair* on July 3rd, 1909, linked to a patent (Bates et al., 1912) for a system whereby a cinematograph is targeted as a shooting range (see Figure 4). The piece is worth quoting at length given the unbelievability of the suggestion: “The big-game hunter can crouch behind an armchair, and, resting the muzzle of his gun on the chair, watch the screen and wait. A scene flickers before him: the room fades away, and he is in Uganda “on safari” lion-hunting. The lion appears. The armchair big-game hunter can choose his moment to shoot and the spot where he shot will prove fatal. He fires. The picture stops, and though the lion does not drop, he remains in the position in which he stood when the shot was fired. But on the screen the bullet has made its mark, and so the hunter knows where he has shot his quarry. All the joys of deer-stalking, the tremendous thrill of the elephant and rhinoceros shooting, and the delight of bear hunting can be indulged in without going in search of big game.” This form of interactive entertainment appears to have enjoyed a short spurt of popularity in the years before the First World War (see Cowan, 2018). It would presumably also have involved tactile feedback from the recoil of the gun when fired, and hence perhaps deserves to be counted as another example of participatory cinema (or gaming) that included some form of tactile feedback.

### The Ghost Train and Other Fairground Attractions

The Ghost Train rides that appeared on the fairground and in theme parks over the last century often incorporated invisible “danglers” (i.e., pieces of dangling string, etc.) to surprise those on the ride when they unexpectedly feel their touch (see Spence, 2024, for a review). This, then, hints at the use of unexpected tactile stimulation to induce a surprise reaction in an entertainment context. One of the challenges with introducing surprising tactile stimulation is the irritation that some people may feel (even with such a low-tech solution). As Gilbert Chadwick put it when talking about the two-storey Ghost Train that he was building: “We found if you walk into something in the dark you can stop yourself fast. Say like a bit of string hanging up, you stop, bump! If you’re in the Ghost Train the strings hit you, and then its [sic] passed. If you walk into it you rip it down in temper, because it's fear. But if you ride you get the horror and the ride.” (cited in Dallas, 1971, p. 161). Note that the fairground can in some sense be seen as specialising in bodily entertainment (Lynn, 2006; Spence, 2022a, 2022b). As Walton (2007, p. 48) notes: “If you want a good shaking, come and go on the whip on the Pleasure Beach at Blackpool, A ride that delivers vibration.” More recently, researchers working in the United States have started to assess the experiential value of adding a haptic element to a haunted house experience (Clepper et al., 2020, 2022).

### The Sensorama Stimulator

**Figure 5.**
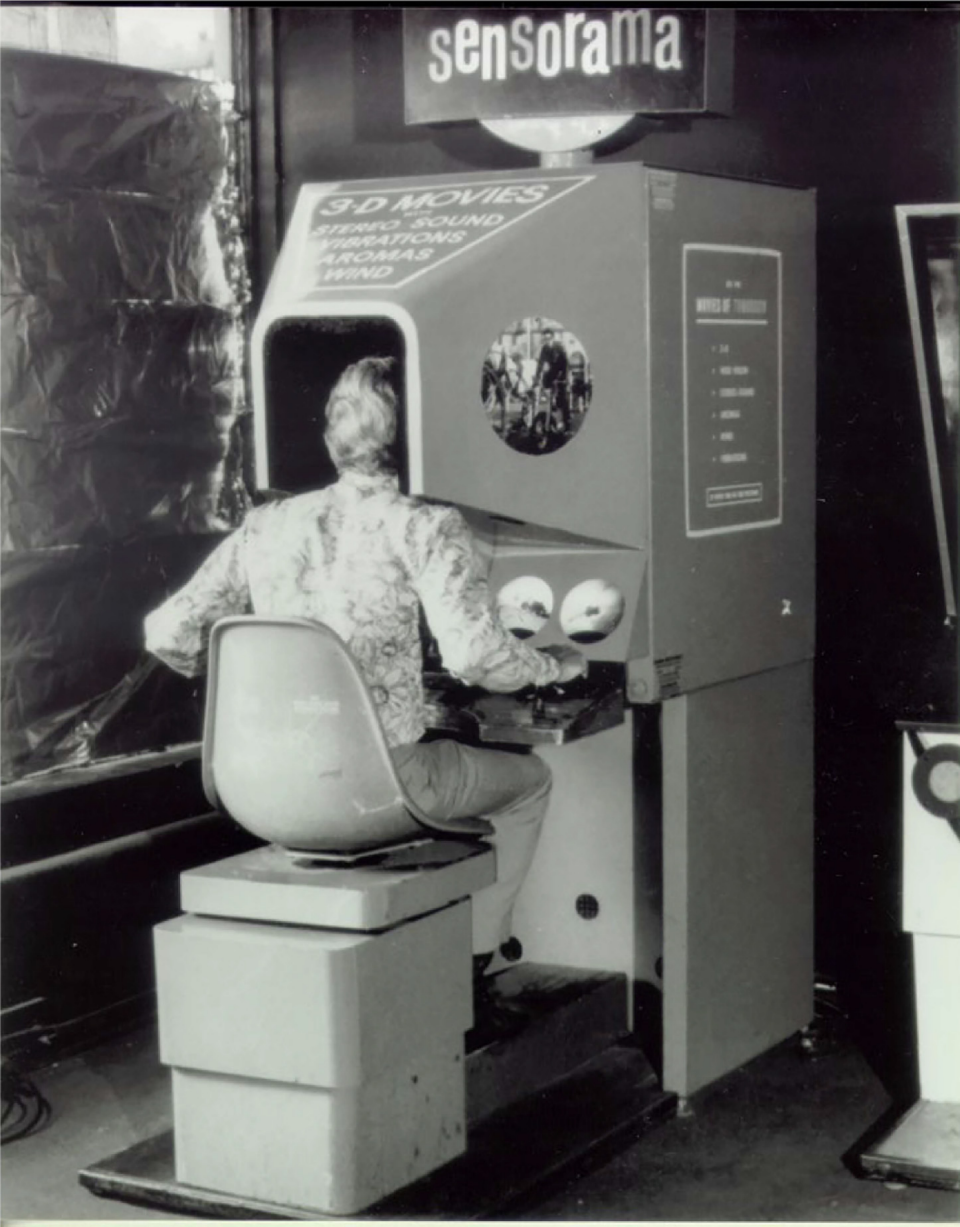
Morton Heilig’s Sensorama simulator (Heilig, 1962).

One of the earliest attempts to create a simulation involving stimulation presented to various different body parts and involving multiple senses at the same time was “Sensorama,” developed in 1957 (and then patented) by the man who is acknowledged as “the father” of VR, Morton Heilig. This device consisted of a machine in which the user was presented with 3D images, smells, stereo sound, wind, and vibrations (see Heilig, 1962, 1992). A small number of films (or rather “simulations”) were made especially for Sensorama. In one, the user experienced various sensations associated with a motorcycle ride through Brooklyn. The sense of presence was meant to be enhanced by the wind blown through the user's hair, by presenting the sounds and smells of the city, and by simulating bumps in the road by means of a vibrator mounted in the chair (see Figure 5). One might think of this as an early version of the “rumble strip” (see Parisi, 2018; Paterson, 2007). A closer inspection of the original patent application (Heilig, 1962, p. 7) reveals that the other simulations that were envisioned all involved travel too: “vibrations of relatively small amplitude are sufficient to create illusions of reality during such scenes as a bobsled ride, a landing aircraft touching a runway, a train ride, etc. However, by switchable programming, the vibrators may be activated unevenly and may also provide sensations of bumps or impacts.” (The idea of multisensory VR was then famously picked up again by Ivan Sutherland with his ideas around the ultimate display [Sterling, 2009; Sutherland, 1965].) In other words, it was only ever tactile stimulation associated with simulated movement that was felt in the Sensorama stimulator. Although the Sensorama simulator clearly represents an example of individual (i.e., rather than the public, or shared) augmentation of the tactile channel, it nevertheless fits as an updated version of the immersive multisensory travel experiences that were first popularised by Hale's Tours, and the *Maréorama*, at the start of the 20th century.

## 4D Cinema and the Theme Park

The virtual travel experiences offered previously by the *Maréorama* multisensory experience, Hale's Tours, and Heilig's Sensorama have continued in the form of motion-simulation rides such as *Trip to the Moon* (Disneyland 1955) and *Impressions of Speed* (Brussels’ International Exposition, 1958; Rabinovitz, 2006). Subsequent virtual travel experiences include the *Tour of the Universe* at Toronto's CN Tower in 1986, *Star Tours* at Disney theme parks; *Body Wars* (1989) at the Epcot Center in Orlando; *The Indiana Jones Adventure: Temple of the Forbidden Eye* (1995) at Disneyland in Anaheim California; and Universal Studios parks *Back to the Future*—*The Ride* (1991). While the majority of these motion platform-type rides are now to be found in theme and amusement parks, a few can also be found in other venues such as *In Search of the Obelisk* (1993) at the Hotel Luxor, Las Vegas. What is seemingly common to all these movable platform rides is that the vestibular/tactile stimulation that the technology delivers was only ever intended to align with the motion shown on screen. Although one might therefore be tempted to criticise this as a merely pleonastic addition of an extra sensory cue (namely movement of the body; cf. Banes, 2001), it is important to note that there is something altogether engaging when it is one's own body that moves (see Spence, 2022b). It can also be argued that by simulating motion, the fourth wall is effectively broken, and the audience feels that they are, in some sense, part of the action that is displayed on screen, rather than being merely passive observers in a stationary auditorium. In this sense, the augmentation of entertainment through bodily movement is qualitatively different from other forms.

## Testing the Impact of Tactile Stimulation in the Cinema

Having highlighted the limitations with the research on technology-controlled touch being incorporated in the context of cinema, it only remains for us to provide some suggestions concerning how such research should be conducted in the future.

At the outset, preregistration might be beneficial in order to avoid the file drawer problem (Rosenthal, 1979; Willroth & Atherton, 2024). The use of multiple short film clips presented either with (or without) haptic augmentation would seem like a sensible research strategy (Danieau et al., 2014). At the same time, however, one should also consider the multiple uses of haptic augmentation in the context of cinema (cf. Banes, 2001). Additionally, there may be salient challenges (in terms of time and cost) around adding or trying to tactile/haptic stimulation to a film that was not created with a haptic track. As such, it might be easier to find a film that had originally been recorded with a haptic track (which, as we have seen, will most likely be some form of rumble, or vibrotactile, stimulation). One approach would be to break the various scenes where touch is stimulated, and then either present the clip with (or without) tactile accompaniment. In terms of the response measure, immersion in, and enjoyment of, the action would appear to be sensible questions to ask (see Steele, 2023). It might also be relevant to enquire how much of a price premium participants would be willing to pay for such haptic augmentation (be it when buying a cinema ticket, or perhaps in the context of filmic content that is increasingly being viewed on an individual's mobile device). Once the basic effect of tactile/haptic augmentation had been quantified, subsequent research could also help by assessing the viewer's sensitivity to asynchrony in terms of the matching vibrotactile stimulation (cf. Spence et al., 2003; Vatakis & Spence, 2006).

It is noticeable how the majority of tactile stimulation has been of the rumble type (i.e., vibration). It should be noted that there are various other tactile channels including thermal (as predicted would be stimulated by Heilig, 1992), pressure, etc. However, the only digitally controlled non-vibration tactile stimulation that is currently available on the market is Sensiks (https://www.sensiks.com/). This can deliver both thermal cues as well as wind. And while some bespoke short-format content has been released for this system, we are not aware of any widespread entertainment examples that involve thermal stimulation. Wind effects have appeared occasionally (as mentioned in several of the earlier examples), though this presumably will activate several somatosensory systems simultaneously.

## Conclusions

As highlighted by this narrative historical review, there has long been interest in augmenting cinematic and other forms of public entertainment by means of tactile and/or bodily (i.e., vestibular) stimulation (see Table 2 for a summary). The early history of touch (or haptics, as it is sometimes called) and other forms of bodily stimulation (e.g., as in the case of motion platforms) in the entertainment context is critically reviewed, with a focus on early cinema, as well as other examples of immersive multisensory VR travel experiences. Indeed, as the examples discussed here so clearly highlight, interest in the delivery of immersive multisensory entertainment experiences has a much richer history than is sometimes realised (as evidenced by the existence and popularity of multisensory experiences such as the *Maréorama and Hale's Tours*)*.*

Various challenges that have limited the introduction of such additional channels of sensory stimulation are identified, including the technical, financial, cognitive, and creative (see Table 3, for a summary). Taken together, the existence of such considerations helps to explain why it is that despite the early interest in “the feelies” (e.g., by Aldous Huxley, in his novel *Brave New World*; Huxley, 1932), touch-enhanced cinema and storytelling have never really caught on in the mainstream in the way that, say, the talkies so obviously did following the introduction of sound into cinema in the early decades of the 20th century (Kehr, 2010). At the same time, however, identifying the potential successful use cases that have emerged from previous attempts to augment public entertainments with tactile/bodily stimulation will likely provide useful guidelines for the future tactile augmentation of home entertainment (see Clark, 2018).

The entertainment business is huge (Cheok et al., 2014; Harrington, 2017), and the market for home entertainment is growing rapidly (Anon, 2023). According to one recent industry report: “The Global Home Entertainment System Market is estimated to be USD 271.03 Bn in 2023 and is expected to reach USD 388.01 Bn by 2028, growing at a CAGR of 7.44%. The use of multiple electronic entertainment devices to create and reproduces [sic] a movie theatre experience and mood in your home is called Home Entertainment System” (Anon, 2023). As such, it becomes increasingly relevant to consider the future possibilities associated with touch-enabled home entertainment, as consumers increasingly experience much of their digital content on their smartphones and other mobile devices (see Gallace & Spence, 2014). This change in the way in which people consume multisensory entertainment means, on the one hand, that vibrotactile tactile stimulation is likely to become more common, while whole body movement is impractical in the home setting. Hence, the opportunities and challenges for haptically enabled technology in the home are somewhat different from those associated with traditional public entertainment contexts (as reviewed here). It is interesting to see who (if anyone) is currently operating successfully in the space of touch-augmented entertainment to get a sense of what might one day be possible (either digitally or with actual physical touch; see, e.g., Armitage & Ng, 2013, on the use of haptic technology to augment opera performance; and Clark, 2018). At the same time, however, a number of key challenges/barriers to the incorporation of touch (by which we mostly mean digital vibrotactile touch these days) are the same in the home entertainment context as have been identified here in the case of public entertainment. An additional challenge, however, that is largely restricted to home entertainment is legal in nature, given the many patents for digital touch that are both held, and actively enforced by the likes of the Immersion Corporation (as discussed at length, elsewhere; e.g., see Murdey, 2006; Parisi, 2018).

Even if the technological, financial, cognitive, ethical/artistic and possibly also legal (in the context of home tactile stimulation) challenges could somehow be overcome, there remains the fundamental question of what, exactly, the goal of stimulating the skin and/or body might be, in an entertainment context. It is by no means obvious that immersion in the experience will necessarily be enhanced, nor is a technology-mediated emotional response likely to be elicited either (see Spence, 2022c). The danger is that tactile augmentation is merely pleonastic in nature (Banes, 2001), meaning that one simply presents to the skin what is already visible or audible to the audience through their other sensory channels. Pleonastic tactile augmentation, that is, merely reproducing what is seen on screen or heard through vibrating the back of cinema-goer's chair is unlikely ever going to be a price that is worth paying. It should, though, be borne in mind that the price of technology tends to decline as the years since its introduction pass. At the same time, however, prices tend to decline more for those technologies that have widespread uptake in the market, and there is little evidence that the desire for touch-enabled film entertainment has really caught on in the mainstream.

Frost (2006, p. 466) writes that: “It seems fitting that the feelies never made it to the big screen, for any effort to depict them cinematically would necessarily dilute their polysensual dimensions. Almost 75 years after *Brave New World* was published, the movies are still not “stereoscopic,” and commercial films are still incapable of making an audience feel the hairs on a tigerskin rug. The irony is that the feelies, an incarnation of pure physical experience in *Brave New World*, cannot themselves be embodied or represented visually. Despite Huxley's anxious predictions about cinema bringing on the end of real culture, his own most fearsome representation of cinema's future, the feelies, only exists through the written word. Literature, not film, remains the medium most capable of imagining and representing the most spectacular of pleasures.”

As yet, there have been very few cases where tactile stimulation has been used to convey action/information that cannot also be conveyed visually and/or auditorily (though see Clark, 2018, for one such example, from a short story/experience involving an Ouija board). The often stated promise that engaging more senses will necessarily lead to increased engagement/immersion (e.g., Nakamura et al., 2024) has proved challenging to demonstrate outside the realms of moveable platforms (as in VR rides). Furthermore, given that the tactile experience of the audience is never going to precisely match what is being depicted on screen (e.g., either in terms of viewpoint from which the haptic action is experienced or in terms of the realism of what is being depicted by means of tactile, as opposed to bodily, stimulation), there is a danger that bodily stimulation might serve to remove people from the action (and/or distract them). Furthermore, should more realistic forms of digital tactile stimulation ever be developed then it may well give rise to an “uncanny valley of haptics”: this the phenomenon where increasing the realism of an interaction increases the feeling of unease as its representation becomes more, but never quite fully, realistic (see Berger et al., 2018; Desnoyers-Stewart et al., 2024). One of the other problems that have been identified in the context of using digital touch to augment entertainment is that the perspective of tactile stimulation may be misaligned (Zhang et al., 2022), further distracting from the intended enhanced immersion in the multisensory experience.

Ultimately, therefore perhaps the most promising use for tactile/bodily stimulation relates to its potential ability to break the “Fourth Wall” normally separating the audience from the action seen and/or heard on stage/screen (Fisher, 1997, 2007; see Spence, 2021b, on the use of scent in the context of live performance to precisely achieve this result). Given the limitations that have been identified here, the touch-enabled augmentation of multisensory entertainment would primarily appear to appeal at the sensory level, rather than helping to enhance the storytelling (though see Clark, 2018). This touch-enabled augmentation might still be at an early stage of incorporating new technology into the storytelling medium.

## Notes

1. As Frost (2006, p. 449) notes: “Although the feelies are pap for the public, their institutional structure indicates their importance in *Brave New World*… The ‘Feeling Picture’ headquarters comprise 22 floors of the Bureau of Propaganda in London (66), the ‘buildings of the Hounslow Feely Studio’ sprawl over ‘seven and a half hectares’ (62), and the College of Emotional Engineering includes on its faculty ‘professors of feelies,’ a title of considerable status.”

2. Salvador Dalí's *Le cinéma tactile* from the 1930s can also be mentioned here. As Chilton (2019) notes: “A trip to the movies might have been a more bizarre, interactive experience if the Spaniard's concept for ‘Le cinema tactile’ had got off the ground in the 1930s. Dali drew elaborate sketches for his tactile cinema, showing how objects and artefacts would slide out of the back of a cinema seat, enabling viewers to touch items that were being presented on the screen … While he was involved in the production of the movie *L’Age d’Or*, for example, he proposed that cinema-goers would be presented with rubber breasts to fondle while they watched a scene in which a woman was being caressed. He also suggested that hot water should be sprayed on people's hands just as a bidet was being flushed in the movie. Needless to say, this oddball idea was not a viable commercial proposition.”

3. See Rooney and Hennessy (2013) for some limited evidence of the benefit of 3D over 2D film in terms of audience experience.

4. Later that year, Sensurround, a system that had been developed by W. O. Watson and Richard Stumpf of Universal Pictures, was released. This format presented 5–40 Hz tones at 110–120 dB based on control track information on the film. Sensurround movies included *Midway* (1976), *Battlestar Galactica*. *Saga of a Star World* (1978), and *Mission Galactica: The Cylon Attack* (1979); Smight, J. (1976). *Midway* [Film]. The Mirisch Corporation; Colla, R. A., & (uncredited), A. L. (1978). *Saga of a Star World* [Film]. Glen A. Larson Productions, Universal Television. Edwards, V., & II, C. I. N. (1979). *Mission Galactica: The Cylon Attack* [Film]. Glen A. Larson Productions, Universal Television.

5. At the time, there was no practical way to record frequencies below 38 Hz onto a film's soundtrack. Consequently, a pseudo-random noise generator using reference recordings from an actual 1971 Los Angeles-area earthquake created the low-frequency rumble.

6. Guy Marcoux of D-BOX said his firm worked closely with film studios’ motion designers at the postproduction stage to match seat movements frame by frame to the action. There can be 30 separate movements in a frame and the process can take 200–600 hr per film. “We are trying to reflect everything you would feel in real life without crossing that line of being like a theme park ride,” he said. “We are trying to get a mix between motion and emotion” (quoted in Scott, 2013).

7. Presumably to target the following complaint: *“Most designers have gotten to the point in production where the decision is made to hit the viewer with everything they’ve got. The big sounds, the dramatic slam of music from the dead silence, the sudden appearance of the beast. And the kids sit there saying ‘been there … done that … ho hum…”* (Ralph Thomas, quoted in “Nothing to sniff at?,” 2002).

8. Version of electric shock-based entertainment games still exist. Just take *The New Addams Family Electric Shock Machine* (Nova Productions, 1999; see also Costa, 1988).

9. Although it was preceded by other similar, if slightly less impressive multisensory entertainments. For instance, Hernández Barbosa (2023, p. 360) notes that: “In France, panoramas which included moving stalls, and which were as spectacular as the rotunda, were introduced only in the early 1890s with the panorama *Le Vengeur*, the creation of the painter Henri Poilpot. Situated at the rotunda of the Champs-Elysées, it evoked a naval battle seen from a ship that rocked and was propelled by a system of pistons, and thus simulating an action placed within a set timeframe (Michaux, 1999, p. 65).”
